# Effects of Salivary Oxidative Markers on Edentulous Patients’ Satisfaction with Prosthetic Denture Treatments: A Pilot Study

**DOI:** 10.1371/journal.pone.0151605

**Published:** 2016-03-17

**Authors:** Chia-Huang Chang, Chang-Yu Lee, Sheng-Wei Feng, Nae-Fang Miao, Pei-Huan Lin, Che-Tong Lin, Shin-Han Tsai, Yung-Kai Huang

**Affiliations:** 1 College of Public Health and Nutrition, Taipei Medical University, Taipei, 110, Taiwan; 2 School of Oral Hygiene, College of Oral Medicine, Taipei Medical University, Taipei, 110, Taiwan; 3 Division of Periodontics, Department of Dentistry, Taipei Medical University Hospital, Taipei Medical University, Taipei, 110, Taiwan; 4 School of Dentistry, College of Oral Medicine, Taipei Medical University, Taipei, 110, Taiwan; 5 School of Nursing, Taipei Medical University, Taipei, 110, Taiwan; 6 Division of Prosthodontics, Department of Dentistry, Taipei Medical University Hospital, Taipei, 110, Taiwan; Navoadaya Dental College and Hospital, mantralayam Road, INDIA

## Abstract

**Objectives:**

The purpose of this study was to assess relationships among periodontal conditions, salivary antioxidant levels, and patients’ satisfaction with their prostheses.

**Methods:**

This study was conducted at the Division of Prosthodontics, Department of Dentistry, Taipei Medical University Hospital. The periodontal condition of patients was based on an assessment of the plaque index (PI) and gingival index (GI). The pH value, flow rate, and buffer capacity of the saliva were estimated. The salivary total antioxidant status (TAS) and superoxide dismutase (SOD) level were also determined. Patients’ satisfaction with prosthetic treatments was evaluated using the Chinese version of the short-form Oral Health Impact Profile (OHIP-14C). A multivariate regression model was used to determine whether patients’ satisfaction with prosthetic treatment was affected by their oral health status.

**Results:**

In total, 35 edentulous patients were recruited. In the Spearman correlation analysis, salivary pH (*r* = -0.36, *p* = 0.03) and the buffer ability (*r* = -0.48, *p*<0.01) were associated with OHIP-14C scores. In the multivariate analysis, patients who had a higher GI also had a higher score of physical disabilities (β = 1.38, *p* = 0.04). Levels of SOD increased with the scores of psychological discomfort (β = 0.33 U/g protein, *p* = 0.04).

**Conclusions:**

This study suggested that both the GI and SOD levels were associated with patients’ satisfaction with prosthetic treatments. To the best of our knowledge, this is the first study to elucidate the relationship between OHIP scores and salivary oxidative markers in edentulous patients.

## Introduction

With increasing life expectancies, aged populations in developing countries are increasing year by year. Oral health is recognized as an essential and integral component of the general health and well-being of older persons [1]. Tooth loss is a major concern among older adults. Partial and complete edentulism can lead to impaired masticatory function, an unhealthy diet, social disability, and a poor health-related quality of life (QoL, HRQoL) [2]. It is noteworthy that high prevalences of edentulism are found among elderly populations [2–5].

The oral HRQoL (OHRQoL) is a multidimensional concept with comprehensive evaluations of oral health and function, social and emotional well-being, environment, and treatment expectations [6, 7]. OHRQoL measures were developed and are applied in the clinical practice of dentistry and dental treatments. The Oral Health Impact Profile (OHIP) is a widely used instrument with proven excellent validity and reliability [8–10]. The OHIP is applied to determine the treatment efficacy, patient satisfaction, dental esthetics, and prosthetic rehabilitation and was translated and adopted in many countries [11–14].

In partially and fully edentulous patients, treatment with oral prostheses can improve their masticatory efficiency, aesthetics, and psychological benefits. Nevertheless, it was noted that patient dissatisfaction in relation to physical, functional, and psychological conditions still existed after treatment [11, 15, 16]. Early studies indicated that the prognosis of oral prostheses was closely related to oral hygiene and the oral status of patients [17, 18]. Decreased masticatory performance resulting from partial or complete dentures may influence the dietary food selection and nutritional intake [19–21]. Malnutrition can lead to antioxidant changes [22]. Our previous study also showed that salivary antioxidants are prognostic biomarkers of periodontal treatment [23]. However, associations among oral hygiene, physical properties of the saliva, and prosthetic prognoses are rarely mentioned. The aim of this study was to explore relationships among periodontal conditions, properties of the saliva, and patients’ satisfaction with prosthetic treatments.

## Materials and Methods

### Subjects recruited

Participants in this study were enrolled from Division of Prosthodontics, Department of Dentistry at Taipei Medical University Hospital between November 2011 and October 2012. In this study, the occlusal supports were evaluated using the Eichner Index which was used to present the posterior occlusal support zones. Eichner Index was divided into three main groups: A, occlusal contacts in four posterior support zones; B, occlusal contacts in three to one posterior support zone(s) or only in the anterior region; C, no occlusal contacts [24]. After assessing the prosthodontics need of patients by dental prosthetics, patients who received complete dentures (CDs), removable partial dentures (RPDs), or fixed partial dentures (FPDs) were recruited for this study. All participants provided written informed consent before the questionnaire interview and salivary specimen collection. The Research Ethics Committee of the Taipei Medical University Joint Institutional Review Board (Taipei, Taiwan) approved this study, and the study complied with the World Medical Association *Declaration of Helsinki*.

**Fig 1:**
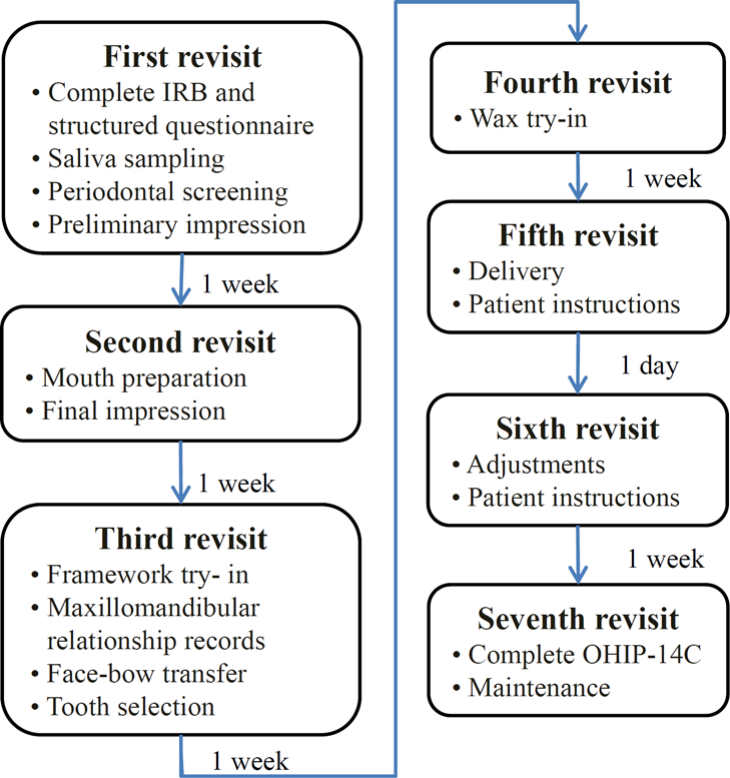
Flowchart of data collection and the prosthetic process.

In total, 35 participants were recruited. There were 5 patients treated with FPDs, 17 patients with RPDs, 4 patients with CDs, 3 patients with FPDs and RPDs, and 6 patients with CDs and RPDs. The procedures for data and specimen collection are shown in Fig 1. Before prosthetic treatment, each participant completed a structured questionnaire that collected sociodemographic characteristics (age, weight, height, and educational level), lifestyle factors (smoking, alcohol consumption, and betel nut chewing), and personal and family disease histories. Oral hygiene and periodontal conditions were based on assessing the plaque index (PI) and gingival index (GI) scales, physical properties of the saliva, and the oral antioxidant capacity.

### Collection of clinical indices

Clinical indices (PI and GI) of subjects were evaluated in this study. Measurement of the PI was based on a record of both soft debris and mineralized deposits on 4 surfaces of a tooth (buccal, lingual, mesial, and distal), and a score of 0~3 was given [25]. Scores of the PI were defined as follows: 0, no plaque; 1, a film of plaque may be seen in situ only after application of disclosing solution or by using a probe on the tooth surface; 2, moderate accumulation can be seen with the naked eye; and 3, an abundance of soft matter within the gingival pocket and/or on the tooth and gingival margin.

The GI was an assessment of inflammation of periodontal tissues, and a score of 0~3 was given [26]. GI scores were classified as follows: 0, no inflammation; 1, mild inflammation; 2, moderate inflammation; 3, severe inflammation.

### Physical properties of the saliva

The pH value, flow rate, and buffer capacity were determined using a Saliva-Check kit (GC Corporation, Tokyo, Japan). Participants were instructed not to smoke, consume food or drink, brush the teeth, or use a mouthwash for at least 1 h prior to saliva sampling. Saliva samples were collected by chewing a piece of wax for 5 min. The flow rate was calculated by the quantity of saliva at 5 min. The pH value and buffer capacity were measured using a pH test strip and buffer test strip, respectively. Normal physical properties of the saliva were set as follow: pH of ≥ 6.8, a flow rate of > 5.0 ml/5 min, and a buffering ability of ≥ 10.

### Antioxidant determination

The saliva sample was mixed with 20 μL of a protease inhibitor and centrifuged at 2000 *g* for 15 min. After centrifugation, the supernatant solution was separated and extracted for analyzes of the total antioxidant status (TAS) and superoxide dismutase (SOD). The TAS and SOD were respectively examined using a Total Antioxidant Status kit (Fortress, Antrim, UK) and Superoxide Dismutase Kit (Randox, Crumlin, UK) in accordance with the manufacturer’s instructions.

### OHIP

Patients’ satisfaction with prosthetic treatments was estimated by the Chinese version of the short-form OHIP (OHIP-14C) [13]. Patients completed the OHIP-14C at 1 week after treatment. The OHIP-14C was used to assess 7 dimensions of patients’ satisfaction, including functional limitations, physical pain, psychological discomfort, physical disabilities, psychological disabilities, social disabilities, and handicaps. Each item of the OHIP-14C was determined on a 5-point Likert scale (0 = never, 1 = hardly ever, 2 = occasionally, 3 = fairly often, 4 = very often). The OHIP-14C score was a summation of 14 individual item scores.

### Statistical analyses

Data analysis was carried out with SAS software (vers. 9.3). Statistical significance was set at *p*<0.05. The internal consistency of the OHIP-14C was assessed by Cronbach’s α. Correlations among sociodemographic characteristics, periodontal conditions, salivary properties, and OHIP-14C scores were initially explored using Spearman’s correlation. Potential confounders, including age, sex, and the body-mass index (BMI), were mainly considered. A multivariate regression model was used to determine whether patients’ satisfaction with prosthetic treatments was affected by periodontal conditions or salivary antioxidant levels.

## Results

Among 35 participants, the average age was 69 years and the average BMI was 23.9 kg/m^2^. More than 40% (*n* = 14) of patients were classified as overweight or obese. Percentages of participants who smoked, consumed alcohol, and chewed betel nut were 34.3%, 28.6%, and 5.7%, respectively. The majority (54.3%) of subjects were classified into group B of the Eichner index. Almost half (47.1%) of patients had hypertension. Median concentrations of SOD and TAS were 1.87 U/g protein and 5.22 mmol/g protein, respectively. There were no significant differences in SOD or TAS between the groups with respect to demographic characteristics, such as sex, BMI, smoking, and betel nut chewing (Table 1).

**Table 1:** Sociodemographic characteristics of participants.

	Number of subjects	%	SOD (U/g protein)	*p* value	TAS (mmol/g protein)	*p* value
			Median (SD)		Median (SD)	
Total	35		1.87 (2.49)		5.22 (3.43)	
Sex						
Male	18	51.4	1.55 (2.63)	0.15	5.36 (3.63)	0.18
Female	17	48.6	2.24 (2.34)		3.74 (3.15)	
Body mass index (kg/m^2^) ^#^						
<18.5	3	8.8	1.98 (0.95)	0.59	3.74 (1.16)	0.43
18.5–25	17	50.0	1.87 (1.40)		5.25 (3.19)	
≥25	14	41.2	2.06 (3.41)		5.59 (3.87)	
Years of schooling						
≤6	9	25.7	1.41 (1.81)	0.69	3.51 (4.05)	0.42
7~12	15	42.9	1.81 (3.25)		5.22 (3.20)	
>12	11	31.4	2.11 (1.74)		5.27 (3.27)	
Smoking						
No	23	65.7	1.98 (2.15)	0.59	3.84 (3.27)	0.12
Yes	12	34.3	1.55 (3.13)		5.36 (3.48)	
Alcohol consumption						
No	25	71.4	2.24 (2.74)	0.03	5.22 (3.50)	0.80
Yes	10	28.6	1.46 (0.60)		5.14 (3.40)	
Betel nut chewing						
No	33	94.3	1.87 (2.44)	0.60	5.22 (3.39)	0.83
Yes	2	5.7	4.14 (3.92)		3.87 (5.48)	
Eichner Index						
A	6	17.1	3.11 (2.05)	0.22	4.26 (2.55)	0.32
B	19	54.3	1.81 (3.01)		5.86 (3.96)	
C	10	28.6	1.39 (1.15)		3.63 (2.72)	
Medical disease^#^						
Hypertension	16	47.1	1.89 (2.58)	0.88	3.96 (3.58)	0.28
Diabetes mellitus	8	23.5	2.17 (3.07)	0.43	3.80 (3.98)	0.33
Hyperlipidemia	4	11.8	1.89 (2.82)	1.00	5.00 (2.28)	0.98
Others	7	20.6	2.11 (1.00)	0.75	4.09 (2.55)	0.35

SOD, superoxide dismutase; TAS, total antioxidant status; SD, standard deviation.

^#^ One participant had a missing value.

*p* values were determined by the Wilcoxon rank sum test or Mann-Whitney U-test.

**Fig 2:**
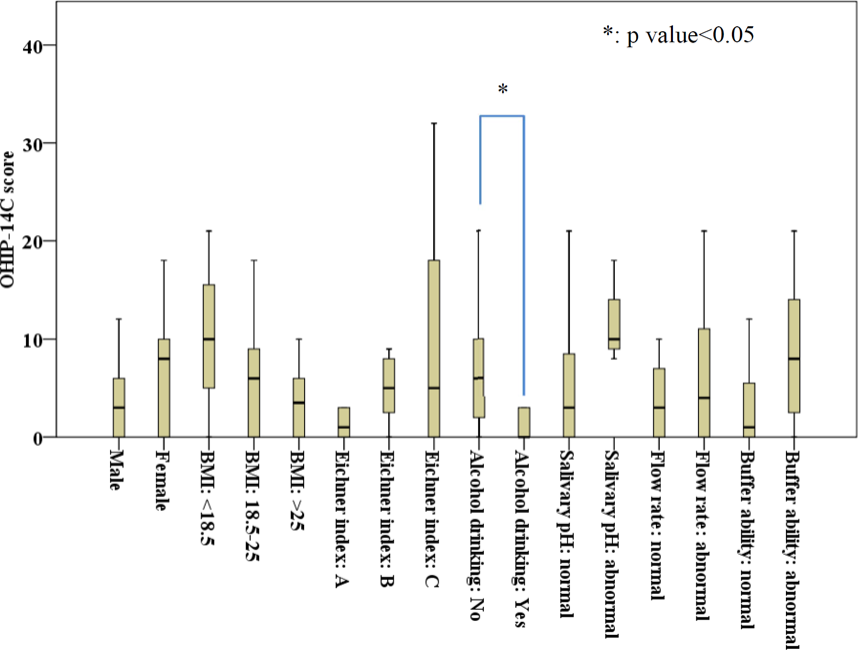
OHIP-14C score distributions by demographic characteristics and salivary physical properties.

Cronbach’s α of OHIP-14C ranged 0.67~1.00 for the 7 dimensions. The mean OHIP-14C score of participants was 6.69 (range, 0~32). Mean scores of the 7 dimensions are shown in Table 2. No statistically significant difference existed for OHIP-14C scores between gender. The were no statistically significant difference in OHIP-14C scores among sex, BMI, Eichner index, or salivary physical properties. Participants who did not drink alcohol had significantly higher OHIP-14C scores compared to those who did consume alcohol (Fig 2).

**Table 2:** Internal consistency and mean scores of the OHIP-14C among participants.

	Cronbach’s α	Mean	Range
OHIP-14C	0.92	6.69	0~32
Functional limitations	0.67	1.63	0~5
Physical pain	0.92	1.57	0~5
Psychological discomfort	0.97	0.69	0~8
Physical disabilities	0.67	0.77	0~5
Psychological disabilities	0.92	1.14	0~8
Social disabilities	0.99	0.54	0~6
Handicaps	1.00	0.34	0~4

The mean salivary pH, flow rate, and buffer capacity were 7.29, 4.29 ml/5 min, and 6.90, respectively. Salivary properties of participants that were classified as normal by pH, flow rate, and buffer ability accounted for 91.4%, 34.3%, and 45.7%, respectively. The buffer ability was significantly associated with the pH (*r* = 0.70, *p*<0.01) and flow rate (*r* = 0.49, *p*<0.01). The mean PI was 0.84, and the mean GI was 0.65. There were significant correlations of PI with GI (*r* = 0.42, *p* = 0.03) and SOD (*r* = 0.40, *p* = 0.03) (Table 3).

**Table 3:** Spearman’s correlation among salivary properties, periodontal conditions, and antioxidant levels.

	pH	Flow (mL/min)	Buffer capacity	Plaque index	Gingival index	TAS (mmol/g protein)	SOD (U/g protein)
Mean (SD)	7.29 (0.36)	4.29 (2.84)	6.90 (4.30)	0.84 (0.45)	0.65 (0.56)	5.05 (3.43)	2.69 (2.49)
Q1, Q3	7.2, 7.6	2.0, 5.0	2.0, 12.0	0.50, 1.01	0.38, 0.75	2.90, 7.69	1.14, 3.47
	**Spearman’s correlation coefficient**						
pH	1	0.33	0.70**	-0.21	-0.29	-0.07	-0.20
Flow		1	0.49**	-0.10	-0.25	0.22	0.14
Buffer capacity			1	-0.14	-0.35	0.09	-0.27
Plaque index				1	0.42*	-0.12	0.40*
Gingival index					1	-0.22	-0.03
TAS						1	0.18
SOD							1

SD, standard deviation; TAS, total antioxidant status; SOD, superoxide dismutase.

* *p*<0.05

** *p*<0.01.

In Spearman’s correlation analysis, salivary pH (*r* = -0.36, *p* = 0.03) and the buffer ability (*r* = -0.48, *p*<0.01) were associated with OHIP-14C scores. After adjusting for age, sex, the BMI, alcohol consumption, and salivary physical properties, patients who had a higher GI also had higher scores of physical disabilities (β = 1.38, *p* = 0.04). The level of SOD increased with the score of psychological discomfort (β = 0.33 U/g protein, *p* = 0.04) (Table 4).

**Table 4:** Multivariable linear regression model of OHIP-14C scores and periodontal conditions.

Variable	Plaque index		Gingival index		TAS (mmol/g protein)		SOD (U/g protein)	
	β (SE)	*p* value	β (SE)	*p* value	β (SE)	*p* value	β (SE)	*p* value
Model 1 (Functional limitations)	0.19 (0.87)	0.83	0.01 (0.88)	1.00	0.08 (0.06)	0.20	-0.04 (0.15)	0.79
Model 2 (Physical pain)	0.51 (0.63)	0.43	0.71 (0.58)	0.24	0.08 (0.05)	0.13	0.02 (0.13)	0.87
Model 3 (Psychological discomfort)	0.18 (0.83)	0.83	-1.04 (0.86)	0.24	0.02 (0.07)	0.82	0.33 (0.15)	0.04
Model 4 (Physical disabilities)	0.87 (0.64)	0.19	1.38 (0.60)	0.04	0.02 (0.05)	0.78	-0.07 (0.12)	0.57
Model 5 (Psychological disabilities)	0.67 (0.90)	0.47	0.01 (0.96)	0.99	0.00 (0.08)	0.95	0.29 (0.17)	0.09
Model 6 (Social disabilities)	0.74 (0.44)	0.11	0.44 (0.49)	0.38	-0.01 (0.05)	0.93	0.14 (0.10)	0.20
Model 7 (Handicaps)	0.35 (0.39)	0.38	0.45 (0.40)	0.27	-0.02 (0.03)	0.41	0.11 (0.06)	0.10
Model 8 (OHIP)	3.50 (3.05)	0.27	1.95 (3.31)	0.56	0.18 (0.29)	0.55	0.78 (0.65)	0.24

Adjusted covariates: age, sex, body-mass index, alcohol consumption, and salivary physical properties. β, estimated coefficient; SE, standard error; TAS, total antioxidant status; SOD, superoxide dismutase.

## Discussion

Edentulism is an irreversible condition of being toothless. The number of natural teeth plays an essential role in the oral functioning and the oral health status [2, 19, 27–29]. Impaired mastication, in terms of the ability to bite, chew, and swallow, can diminish masticatory performance and efficiency, and further lead to influences on the diet and food selection [27]. Tooth loss accompanied by residual ridge resorption and the loss of alveolar bone can alter one's facial appearance [19, 29]. Speaking ability can also be affected by anatomical changes in the dental status [19]. In this study, improvements in functional limitations (mastication and pronunciation) and physical pain (being uncomfortable to eat and sore spots) were observed after prosthetic treatment.

Conventional prostheses in edentulous patients include CDs, RPDs, and FPDs [30–33]. These treatments differ depending on the number of remaining natural teeth of the patient, chewing efficiency, tooth structures, periodontal conditions, and treatment period. Overall, prosthetic treatments can restore functionality and improve the OHRQoL [12, 14]. Oh et al. indicated that edentulous patients’ satisfaction varied in accordance with the prosthesis type: both the QoL and patients’ satisfaction with FPD and RPD treatments were superior to those with CD treatment [12]. In this study, patients with FPD and RPD treatments had lower mean OHIP-14C scores than those with CD treatment (FPDs vs. RPDs vs. CDs = 1.20 vs. 7.24 vs. 15.50, *p* = 0.08). The majority of participants were satisfied with their prosthetic prognoses. After adjusting for other covariates, patients’ satisfaction with prosthetic treatment was mainly influenced by SOD levels and the GI.

Oxidative stress is regarded as an important factor in periodontal disease. Excessive production of reactive oxygen species (ROS) leads to progressive oxidative damage via the response to periodontal injury and inflammation [34, 35]. A recent study indicated that SOD activity decreased in response to periodontal treatment [36]. Our previous study also demonstrated that an increase in SOD was related to a higher severity of periodontitis and oral health behaviors [23]. SOD is one of the important antioxidants, catalyzes the dismutation of superoxide into oxygen and hydrogen peroxide, and protects cells from oxidative damage [37]. Salivary antioxidants can reflect periodontal tissue conditions and therapy outcomes [23, 36]. In this study, SOD levels among edentulous patients were associated with psychological discomfort. Edentulism can decrease periodontal tissue regeneration and impair the proactive function of the oral mucosa [2]. The importance of salivary antioxidants as prognostic biomarkers of prosthetic treatment should be addressed.

The role SOD plays in periodontal diseases is paradoxical due to different pathological mechanisms. An increase in SOD activity was accompanied by early inflammatory syndrome, while its decrease occurred in response to the pathological progress [34, 36, 38]. Novakovic et al. indicated that patients with current periodontitis had higher SOD levels compared to periodontally healthy subjects [36]. However, Kim et al. reported that patients with severe chronic periodontitis had lower SOD levels than the control group [34]. In this study, high SOD concentrations may have resulted from the severity of oral inflammation among patients with psychological discomfort.

Periodontal diseases remain the major cause of tooth loss. Effective plaque control is the most important step in preventing dental caries and periodontal diseases [1, 39]. Plaque accumulation is mainly attributed to poor oral hygiene and teeth cleaning. Denture wearing has no influence on the deterioration of the periodontal status, such as gingival inflammation, the PI, tooth mobility, or pocket depth [18, 40]. In this study, subjects who were enrolled had no periodontitis or gingivitis before prosthetic treatment. The negative impacts of the GI on physical disabilities should be a concern.

Saliva is an important part of maintaining the oral health: it coats the oral tissues to protect them against thermal and chemical irritation, neutralizes plaque pH after eating, clears food, aids swallowing, and promotes tooth remineralization [41]. Salivary clearance of carbohydrates from the food, acid from plaques, and other substances is influenced by the edentulous environment. Tooth loss can lead to functional deficiencies in the oral mucosa, oral musculature, and salivary glands [2]. Alterations of the salivary flow rate or pH of the saliva may further affect the number of salivary microbes [42, 43]. Findings of this study revealed that normal salivary properties can increase patients’ satisfaction with their prostheses.

Patient satisfaction surveys are a common tool to evaluate healthcare quality and are capable of serving as patient feedback on medical services [44]. The OHIP has been comprehensively used to determine the OHRQoL in various elderly populations [8–10]. The OHIP-14 was developed by extracting 14 items of the OHIP because it was time-consuming during interviews. In this study, participants completed the OHIP-14C in a face-to-face interview after prosthetic treatment. The OHIP-14C's proven good reliability and validity were also supported in this study. SOD could be auxiliary to a determination of OHIP-14C validity.

The variations in antioxidant levels may due to the relatively small sample size and a wide range of ages (interval: 48~91 years). Age plays an important role in changes in salivary antioxidant levels. Age increases with oxidative stress and a reduced antioxidant-protective capacity [45]. With increases in life expectancy, it is noteworthy that a high prevalence of edentulism is found among elderly populations [2]. About 12% of the population in Taiwan is aged ≥65 years, and the prevalence of complete edentulism among the elderly is 26% [46, 47]. Compared to other developed countries, the rate is still high, whereas about 80% of older adults have partial or complete edentulism in Taiwan [47]. Based on a statistical power of 0.8, the required sample size in the current study was 40 patients. The study with 35 subjects had power of 0.74. Although no control group and small sample size were the limitations of this study, the preliminary results can be referred to as a pilot study. This is the first study to explore the effects of salivary oxidative markers on edentulous patients' satisfaction with prosthetic denture treatments.

Although associations among salivary physical properties, periodontal conditions, levels of antioxidants, and patients’ satisfaction exist, there is no denying that patients with poor oral hygiene have inferior health statuses, and this may have biased the results. In addition, risk factors for edentulism have been studied in much epidemiological research, and these consisted of age, gender, education, an unhealthy diet, tobacco use, alcohol use, lifestyle factors, stress, the socioeconomic status, oral health knowledge, dental care, etc. [3, 29, 48–50]. Other factors, including dietary intake, the nutritional status, dental care, and psychological factors were not accurately measured in this study and need to be further accounted for in future studies.

## Conclusions

Results of this study suggest that both the GI and SOD were associated with patients’ satisfaction with prosthetic treatment. To the best of our knowledge, this is the first study to elucidate the relationship between OHIP and salivary oxidative markers in edentulous patients, and the better oral health that edentulous patients had, the more that they were satisfied with their prosthetic prognosis. Further research should focus on the relationship between prosthetic prognosis and patients’ satisfaction with long-term follow-up.

## Supporting Information

S1 File Table and figure dataset. (DBF)
